# Beyond Viral Restriction: The Metabolic Dimensions of Interferon-Stimulated Genes in Antiviral Immunity

**DOI:** 10.3390/v18020160

**Published:** 2026-01-25

**Authors:** Xiaoyu Ding, Libao Liu, Haiming Wei

**Affiliations:** 1 Anhui Key Laboratory of Infection and Immunity, Bengbu Medical University, Bengbu 233000, China; 2025062@bbmu.edu.cn (X.D.); llb15376313979@163.com (L.L.); 2 Basic Medical College, Bengbu Medical University, Bengbu 233000, China; 3 The Key Laboratory of Immune Response and Immunotherapy, School of Basic Medical Sciences, Division of Life Sciences and Medicine, University of Science and Technology of China, Hefei 230001, China

**Keywords:** antiviral immunity, metabolism, protective interferon stimulated genes, pathogenic interferon stimulated genes

## Abstract

Interferon-stimulated genes (ISGs) are classically recognized for their direct antiviral functions, such as viral genome degradation or replication blockade. However, emerging evidence reveals that ISGs orchestrate a broader landscape of host defense by rewiring cellular metabolism. These mechanisms are still not fully understood in the context of antiviral immunity. This review synthesizes recent advances in understanding how ISGs modulate metabolic pathways (e.g., glycolysis, lipid metabolism, amino acids, and nucleotide metabolism) to create an antiviral cellular environment. However, viruses have developed strategies to evade or counteract ISG-encoded proteins, and some even hijack certain ISGs to their advantage. Therefore, we further explore how viruses subvert these ISG-driven metabolic to evade host defenses. Overall, we summarize the current state of knowledge on the interactions between viruses and ISGs and propose that ISGs act as “protective” or “pathogenic” regulators at the dimensions of metabolism, offering new perspectives for targeting host-centered pathways to combat viral infections.

## 1. Introduction

Since their identification in the 1970–1980s, interferon-stimulated genes (ISGs) have been recognized as central mediators of mammalian antiviral innate immunity [1,2,3]. Traditionally, research on ISGs has focused on their direct antiviral effector functions. Following interferon (IFN) signaling, hundreds of ISGs are rapidly upregulated, many of which encode proteins that directly target distinct stages of the viral life cycle [4]. These classical antiviral mechanisms include inhibiting viral entry or uncoating, blocking viral transcription and translation, degrading viral nucleic acids, and disrupting virion assembly and egress [4]. Together, these effectors establish a critical cell-autonomous defense that helps delay viral dissemination and provides essential time for the adaptive immune system to mount a specific response.

However, emerging evidence is expanding our understanding of ISG functions, revealing that roles extend far beyond direct viral confrontation [5,6]. A pivotal, yet still underexplored, dimension of this expanded function lies in the profound reprogramming of host cellular metabolism. Viruses are obligate intracellular parasites that are entirely dependent on the host’s metabolic machinery for energy, biosynthetic precursors, and building blocks to fuel their replication [7]. ISGs are now recognized as key regulators of host metabolic reprogramming [5,8,9]. By remodeling glycolysis, lipid metabolism, and the metabolism of amino acids and nucleotides, ISGs establish a cellular environment that restricts viral propagation. This reprogramming can deplete resources essential for viruses, generate antiviral metabolites, or alter the redox state to favor host defense, collectively constituting an indirect yet sophisticated layer of immune protection.

Intriguingly, this metabolic battlefield is not unidirectional [10]. The dynamic interaction between ISG-driven metabolic alterations and viral replication has resulted in continuous reciprocal adaptation. Viruses have developed sophisticated strategies not only to evade or counteract the antiviral functions of specific ISG-encoded proteins but also to hijack the metabolic reprogramming triggered by the ISG response. Some viruses adeptly exploit these metabolic shifts, turning a presumed host defense mechanism into a condition that benefits viral replication or facilitates immune evasion [11]. Furthermore, dysregulated or persistent ISG activation and the consequent metabolic imbalance can contribute to immunopathology, exacerbating tissue damage and disease severity [12]. Specifically, “protective ISGs” are defined as those that, within a canonical antiviral response, act to restrict viral replication and preserve tissue homeostasis through direct inhibition of the viral life cycle or modulation of host metabolism. In contrast, “pathogenic ISGs” refer to those whose functions are subverted by viruses in specific viral, cellular, or pathological contexts, thereby transforming from host defenders into accomplices for viral benefit or direct drivers of pathology. Therefore, ISGs must be viewed in a metabolic context as dual-purpose regulators, capable of exerting either protective or pathogenic outcomes contingent upon viral species, cellular milieu, timing, and response intensity.

This review synthesizes recent advances at the intersection of ISG biology and cellular metabolism in antiviral immunity. We begin by outlining the induction and multifaceted functions of ISGs, providing a foundation for a detailed analysis of how ISGs reprogram glucose, lipid, amino acid, and nucleotide metabolism to restrict viral infection. Subsequently, we examine the detrimental aspects of this interplay, discussing how viruses exploit ISG-driven metabolic alterations to evade immune responses and how such dysregulation can precipitate immunopathology. By summarizing the current knowledge on these dynamic interactions, we propose that conceptualizing ISGs as “protective” or “pathogenic” immune metabolic regulators offers novel and promising perspectives for developing host-targeted therapeutic strategies against viral infections.

## 2. Induction and Multifunctional Roles of ISGs

The IFN family is conventionally categorized into four major types: type I (IFN-I), type II (IFN-II), type III (IFN-III), and type IV (IFN- IV) interferon [13]. Their signaling is initiated through binding to distinct receptor complexes on the cell surface. IFN-I signal via the interferon-α receptor (IFNAR), composed of IFNAR1 and IFNAR2 subunits, whereas IFN-III engages the interferon-λ receptor (IFNLR), consisting of IFNLR1 and IL-10Rβ [14]. IFN-IV (IFN-υ) utilizes a unique receptor complex formed by IFN-υR1 and IL-10R2 [13]. Both types activate the kinases JAK1 and TYK2, leading to phosphorylation of signal transducer and activator of transcription (STAT) proteins STAT1 and STAT2, which together with interferon regulatory factor 9 (IRF9) form the transcriptional complex ISGF3 [14]. In contrast, the dimeric IFN-γ binds to the interferon-gamma receptor (IFNGR), primarily activating the JAK1/JAK2-STAT1 pathway and resulting in the formation of the gamma-activated factor (GAF) complex [15]. The IFN-υ receptor complex shares the IL-10Rβ subunit with the IFN-III receptor. The conserved “box1” and “box2” motifs, along with potential STAT phosphorylation sites within IFN-υR1, suggest its capability to engage the JAK-STAT signaling pathway [13]. ISGF3 and GAF translocate into the nucleus, where they bind to interferon-sensitive response elements (ISRE) or gamma-activated sequence (GAS) promoter elements, respectively, thereby inducing the expression of hundreds of ISGs [16]. Different IFNs exhibit distinct kinetic properties. IFN-I typically induce a rapid, broad, yet often transient wave of ISG expression, whereas IFN-γ drives a more sustained expression profile, with the set of regulated genes sometimes differing from those induced by IFN-I [4]. IFN-λ can also elicit a persistent ISG response, particularly at barrier surfaces [17]. Notably, the kinetic characteristics of IFN-IV remain unclear.

The four types of IFNs are distinguished by their signaling pathway, expression profiles and kinetic properties, yet they share a core similarity and collectively constitute a multi-layered antiviral defense network. In summary, the IFN system establishes a foundational antiviral defense through a partially shared repertoire of ISG effectors, while leveraging the distinct receptor configurations, spatiotemporal expression patterns, and functional preferences of each type, ultimately forming an immune defense system that is both cooperative and specialized through evolution.

As effector genes induced by type I, II, III and IV interferons, ISGs are well known for their broad antiviral functions, but they also participate extensively in immune regulation, antigen presentation, protein modification, and cell death [18]. While the canonical definition of ISGs centers on their responsiveness to interferons, some ISGs can also be directly and rapidly upregulated through PRR signaling during the initial phase of infection, either prior to or alongside the IFN response. In antiviral defense, ISGs can inhibit viral infection through multiple mechanisms across various stages of the viral life cycle [4,19,20]. For instance, interferon-induced transmembrane proteins (IFITM) and cholesterol 25-hydroxylase (CH25H) restrict viral entry [21,22]. Myxovirus resistance proteins (Mx) recognize viral nucleocapsid-like structures, blocking nuclear import and integration of viral genomes [23]. 2′-5′-oligoadenylate synthetase 1 (OAS1) activates ribonuclease L (RNase L), leading to degradation of cellular and viral RNA [24]. This not only terminates viral replication but also releases additional pathogen-associated molecular patterns (PAMPs), thereby amplifying innate immune responses. The double-stranded RNA-dependent protein kinase (PKR, also known as EIF2AK2) phosphorylates eukaryotic initiation factor 2α (eIF2α), suppressing protein synthesis and halting viral protein production [25]. Furthermore, ISGs such as radical S-adenosyl methionine domain-containing protein 2 (RSAD2) and bone marrow stromal cell antigen 2 (BST2) can inhibit viral particle release during budding [26,27].

Recent studies have increasingly revealed that ISGs also exert antiviral effects by orchestrating host metabolic reprogramming, including modulation of glucose, lipid, amino acid, and nucleotide metabolism [5,28,29]. This expands the functional repertoire of ISGs and adds a metabolic dimension to host antiviral strategies. Notably, ISGs play a dual role during viral infection: while most of them act protectively, certain ISGs can be hijacked by viruses to promote infection or exacerbate inflammatory responses, thereby contributing to viral immune evasion and pathogenesis [30,31]. Therefore, dissecting the metabolic functions of protective versus pathogenic ISGs during infection has emerged as a pivotal focus in the field.

## 3. ISG-Mediated Remodeling of Glucose Metabolism for Antiviral Immunity

Glucose represents the fundamental source of cellular energy [32]. Accordingly, its catabolic breakdown provides essential energy and biosynthetic precursors required for mounting an effective antiviral immune response [32]. Following cellular uptake via glucose transporters (GLUT), glucose is primarily catabolized and transformed through three major metabolic pathways: glycolysis, the pentose phosphate pathway (PPP), and the hexosamine biosynthesis pathway (HBP) [33]. Hexokinase serves as the initial rate-limiting enzyme in glucose metabolism, catalyzing the conversion of glucose to glucose-6-phosphate [34]. The PPP branches off from glycolysis at the glucose-6-phosphate node, with its principal functions encompassing ribonucleotide biosynthesis and the provision of the reducing equivalent nicotinamide adenine dinucleotide phosphate (NADPH) [35]. The key rate-limiting enzyme of this pathway is glucose-6-phosphate dehydrogenase [36]. In comparison to glycolysis and the PPP, only a small fraction (approximately 2–5%) of cellular glucose metabolic flux enters the HBP [12,37]. The rate-limiting step of the HBP is catalyzed by glutamine–fructose-6-phosphate transaminase, which converts fructose-6-phosphate into the pathway end product uridine diphosphate N-acetylglucosamine (UDP-GlcNAc) [38]. As the donor substrate for protein O-GlcNAcylation, UDP-GlcNAc constitutes a core metabolic signaling molecule that enables cells to sense and respond to fluctuations in nutrient availability [38].

Glucose metabolism represents a critical target of virus-induced metabolic reprogramming. During H1N1 influenza virus infection of the lower respiratory tract, immune cells are rapidly activated to mount a rapid response to viral invasion, leading to a significant increase in local glucose metabolism [39]. This response is accompanied by the upregulation of several key glycolytic enzymes, such as hexokinase 2 (HK2), pyruvate kinase M2 (PKM2), and pyruvate dehydrogenase kinase (PDK) [39]. Even under oxygen-sufficient conditions, cells still prefer to obtain energy through the glycolytic pathway, a phenomenon known as the classic “Warburg effect.” Such metabolic reprogramming not only facilitates rapid adenosine triphosphate (ATP) production but also supplies precursor molecules for processes such as nucleic acid and lipid synthesis, thereby meeting the energy and material demands for both viral replication and host cell proliferation [33]. Interestingly, alveolar macrophages exhibit a distinct metabolic profile, relying more heavily on oxidative phosphorylation and lipid metabolism to maintain homeostasis [40,41]. This metabolic phenotype enables them to effectively perform immune surveillance and clearance functions within the oxygen-rich pulmonary environment, while also ensuring the restoration and maintenance of internal equilibrium during inflammation. Similarly, Epstein–Barr virus (EBV) manipulates host metabolism during latent infection in nasopharyngeal carcinoma (NPC) [42]. The viral-encoded latent membrane protein 1 (LMP1) and LMP2 can cooperatively activate the mammalian target of rapamycin complex 1 (mTORC1)–c-Myc signaling axis. Activation of this axis promotes glucose uptake, glycolysis, and protein synthesis, thereby sustaining viral latency and support tumor progression. Furthermore, the frequently mutated NF-κB and epidermal growth factor receptor/phosphatidylinositol 3-kinase (ERBB/PI3K) pathways in NPC further activate mTOR, collectively maintaining this tumor-promoting metabolic state [43].

Glucose metabolism and the antiviral innate immune system exist a broad and profound interaction. It has been reported that RIG-I-like receptors (RLR) signaling drives a metabolic shift from glycolysis to the PPP and the HBP via the adaptor protein mitochondrial antiviral signaling protein (MAVS) [44]. Specifically, peroxisomal MAVS is responsible for redirecting glucose flux into the PPP and inducing IFN-III expression, while MAVS located on mitochondria-associated membranes (MAMs) directs glucose flux into the HBP and promotes IFN-I expression. These findings suggest that MAVS plays a pivotal regulatory role in mediating the crosstalk between RLR signaling and glucose metabolism reprogramming. Glucose-derived metabolic intermediates influence the IFN response through multiple mechanisms, including metabolic regulation, signal transduction, and epigenetic modification. At the metabolic level, glycolytic intermediates such as glucose-6-phosphate, 3-phosphoglycerate, and phosphoenolpyruvate regulate the intracellular NAD^+^/NADH ratio, thereby affecting sirtuin activity and the acetylation status of transcription factors such as IRF3 and IRF7, ultimately regulating IFN-β expression [45,46]. At the signaling level, lactate accumulation driven by the Warburg effect, along with hyperglycemia, disrupts the MAVS/RIG-I complex and consequently suppresses IFN-I production [47,48,49]. In contrast, UDP-GlcNAc enhances IFN-I signaling through modification of IRF5 [12]. Epigenetically, pyruvate-derived acetyl-CoA not only promotes reactive oxygen species generation to potentiate the IFN response but also increases chromatin accessibility through histone acetylation, thereby facilitating the transcription of antiviral genes [33,50].

**Figure 1:**
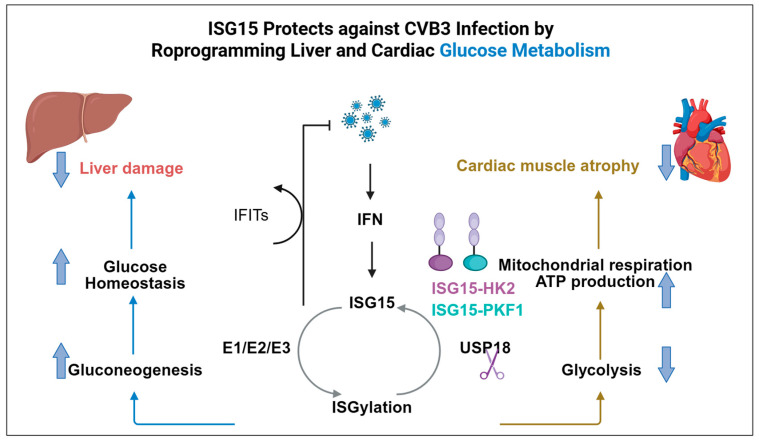
Interferon-stimulated genes (ISGs) inhibit viral infection by regulating glucose metabolism. The model is exemplified by Coxsackievirus B3 (CVB3) infection. Upon IFN stimulation, ISG15 conjugates to target proteins via ISGylation. Early in infection, hepatic ISGylation enhances glucose output and upregulates antiviral effectors. In cardiac tissue, ISGylation conjugates to glycolytic enzymes HK2 and PFK1, preventing infection-induced glycolytic up-regulation. This maintains energy production and protects cardiac function. Targeting the deconjugating enzyme USP18 is a potential host-directed therapy for CVB3 pathology.

Recent studies have revealed that specific ISGs that finely regulate glucose metabolism to meet antiviral demands. As a canonical ISG-encoded protein, the ISG15 can be covalently conjugated to lysine residues of target proteins via an enzymatic cascade involving the E1 activating enzyme (Ube1L), the E2 conjugating enzyme (Ube2L6), and the E3 ligase (Herc5/6), a modification termed ISGylation [51]. As illustrated in Figure 1, in a mouse model of liver injury induced by coxsackievirus B3 (CVB3) infection, ISG15 not only maintains efficient glucose storage in healthy liver tissue but also reprograms hepatic metabolism during early infection to promote glucose production. This process likely provides energy support for the ensuing immune response. Further research revealed that cells lacking the activity of USP18, the specific deISGylating enzyme, exhibit significantly enhanced resistance to CVB3 infection. This finding suggests that inhibition of USP18 may represent a novel host-directed therapeutic strategy against coxsackievirus-related pathologies [52]. In a separate study on a CVB3-induced myocarditis model from the same research group, ISG15 was shown to directly mediate ISGylation of two key glycolytic rate-limiting enzymes, HK2 and phosphofructokinase 1 (PFK1) [5]. This modification effectively inhibits the activity of both enzymes, thereby blocking the upregulation of glycolysis triggered by infection or IFN stimulation. Consequently, this metabolic reprogramming shifts cardiomyocytes from a state highly dependent on glycolysis toward oxidative phosphorylation, enabling them to maintain stronger ATP-generating capacity under the nutrient-limited conditions caused by infection, thereby helping to counteract virus-induced cardiac dysfunction [5]. These findings indicate that ISG15 is not only an important executor of antiviral effects but also a crucial regulatory node in cardiac metabolic homeostasis, capable of optimizing cellular energy metabolism patterns by suppressing glycolysis to adapt to pathological stress. It is noteworthy that USP18 functions not only as a deISGylating enzyme by removing ISG15 modifications but also serves as a key negative regulator of the IFN-I signaling pathway [53,54]. Although ISGylation has been shown to confer antiviral protection in cardiovascular viral infections, research indicates that selectively inhibiting USP18 to enhance ISGylation in this model does not effectively alleviate myocardial inflammation or improve cardiac dysfunction [55]. These findings suggest that therapeutically enhancing ISGylation alone may be insufficient to control inflammation-driven cardiac injury in this model.

In summary, the discovery of ISG15-mediated metabolic regulation reveals that specific ISGs can function as critical nexuses linking immune responses to cellular metabolism, suggesting that immunometabolic pathways may serve as novel therapeutic targets for intervening in virus-associated inflammatory injury. However, the complexity observed in USP18 inhibition indicates that targeting metabolism alone may be insufficient, necessitating future therapeutic strategies to more systematically consider the overall balance of the immune-metabolic network.

## 4. ISG-Mediated Remodeling of Lipid Metabolism During Viral Infection

Lipids represent a structurally diverse class of hydrophobic or amphipathic molecules essential to living organisms [56]. They serve not only as important energy storage forms (such as triglycerides) but also as the core structural foundation of cell and organelle membranes [56]. Furthermore, lipids act as critical signaling molecules involved in cellular communication and as precursors for various bioactive substances, including steroid hormones and vitamin D [56]. Therefore, lipid metabolism plays a central regulatory role in maintaining cellular energy homeostasis, membrane integrity, and signal transduction. Under nutrient-sufficient conditions, acetyl-CoA in the cytosol is carbonylated by acetyl-CoA carboxylase to form malonyl-CoA, which then undergoes iterative reactions catalyzed by fatty acid synthase to produce saturated fatty acids, primarily palmitate [57]. This fatty acid biosynthesis pathway supplies the direct precursors for generating complex lipids such as phospholipids and triglycerides, thereby supporting the expansion and renewal of membrane systems [57,58]. Simultaneously, cells synthesize cholesterol via the mevalonate pathway. Cholesterol modulates membrane fluidity, stability, and permeability and is a key component of plasma membrane lipid rafts microdomains that function as signaling platforms involved in multiple cellular processes [59]. Cholesterol also serves as an essential precursor for the synthesis of steroid hormones, vitamin D, and bile acids [59]. Under energy-stress conditions, cellular metabolism shifts toward catabolism: enhanced fatty acid β-oxidation generates acetyl-CoA, which enters the tricarboxylic acid cycle and ultimately drives sustained ATP production through oxidative phosphorylation, thereby ensuring long-term and efficient energy supply [58,60]. In summary, the cellular lipid metabolic network is a highly integrated and dynamically regulated system. By coordinating anabolic and catabolic pathways, it adapts to varying physiological and stress-induced demands, maintaining the stability of cellular structure and function.

Viral infections often induce reprogramming of host lipid metabolism to provide raw materials and energy for viral entry [61], replication [62,63], assembly [64], and budding [65]. This metabolic reprogramming also shapes immune responses, thereby affecting antigen processing and presentation, cytokine secretion, and immune cell activation [66]. Hepatitis C virus (HCV) closely relies on lipid pathways to promote viral entry, replication, assembly, and secretion. The virus activates signaling pathways of nuclear transcription factors such as sterol regulatory element-binding protein (SREBP) and liver X receptor (LXR), leading to a broad upregulation of lipid synthesis-related genes. Notably, HCV can also induce lipid accumulation and create conditions for the formation of viral replication complexes by suppressing the expression of two key hepatic microRNAs, miR-185 and miR-130b [67]. It has been reported that the target gene network of miR-185 encompasses critical host factors required at multiple stages of HCV infection [67]. These include stearoyl-CoA desaturase 1 (SCD1), which is involved in viral RNA replication [68]; scavenger receptor class B type I (SCARB1), which mediates viral entry and cell-to-cell spread [69]; SREBP2, which regulates lipid synthesis and influences viral replication and assembly [70]; and the low-density lipoprotein receptor (LDLR), which participates in viral entry and replication [61]. In addition, dengue virus infection can promote the synthesis of fatty acids and carbohydrates in macrophages, which not only facilitates viral replication but also activates the host immune response [71]. Elevated cholesterol levels can impair the host antiviral response. The non-structural protein 4 (Nsp4) of porcine reproductive and respiratory syndrome virus (PRRSV) can upregulate the activity of protein phosphatase 2 (PP2), thereby activating 3-hydroxy-3-methylglutaryl coenzyme A reductase (HMGCR)—the rate-limiting enzyme in the cholesterol synthesis pathway [72]. This process increases intracellular cholesterol levels, which in turn inhibits virus-induced IFN-β production and promotes PRRSV replication [72]. Influenza virus infection promotes viral assembly and release by modulating host cholesterol metabolism and lipid droplet formation [73]. Furthermore, influenza virus infection can lead to the reduction in host fatty acid β-oxidation [60], which can be attributed to multiple factors, including a sharp increase in pro-inflammatory cytokines [74], excessive production of nitric oxide (NO), and decreased activity of carnitine palmitoyltransferase II (CPT II) induced by heat stress [75].

**Figure 2:**
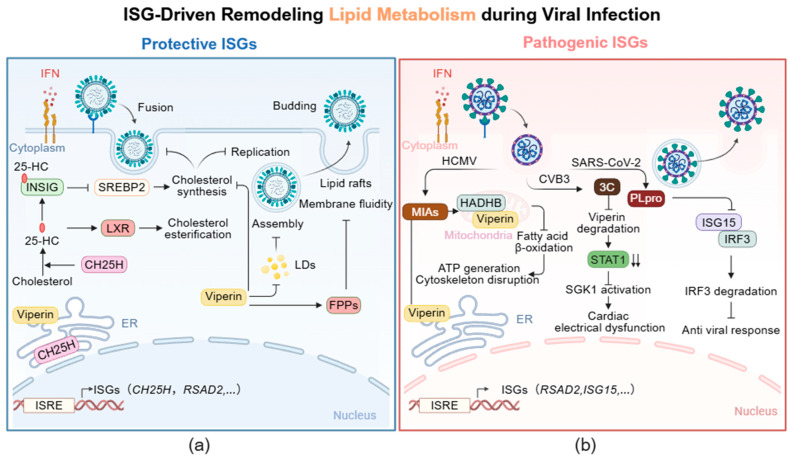
Interferon-stimulated gene (ISG)-driven lipid metabolism reprogramming during viral infection. (**a**) IFN-induced protective ISGs (e.g., *CH25H*, *RSAD2*) restrict viral infection across the viral lifecycle by rewiring cholesterol synthesis and lipid droplet (LD) biogenesis. 25-hydroxycholesterol (25-HC) production was catalyzed by cholesterol 25-hydroxylase (CH25H), which blocks SREBP2 signaling through binding to INSIG (reducing cholesterol production) and acts as a ligand of LXR (promoting cholesterol esterification), jointly impeding viral infection; Viperin (encoded by *RSAD2*) disrupts lipid rafts and reduces membrane fluidity via binding to FPPs, inhibiting viral budding. (**b**) Certain viruses hijack pathogenic ISGs to enhance replication and exacerbate tissue damage. HCMV redirects viperin from the endoplasmic reticulum (ER) to mitochondria through its encoded viral mitochondrial inhibitor of apoptosis (vMIA) protein; once localized to mitochondria, viperin interacts with the mitochondrial trifunctional protein, thereby suppressing fatty acid β-oxidation and reducing cellular ATP production. The 3C protease of CVB3 mediates viperin degradation; subsequently, viperin downregulates STAT1, which in turn activates SGK1 signaling and ultimately induces cardiac electrical dysfunction. The papain-like protease (PLpro) of SARS-CoV-2 cleaves ISG15 conjugates from interferon regulatory factor 3 (IRF3); this modification promotes IRF3 degradation and attenuates IFN-I responses. Solid/faded arrows for positive regulation; blunt-ended lines for inhibition; colored boxes for molecular categories; and double downward arrows (↓↓) for downregulation.

Hosts mount an antiviral defense by regulating ISG-mediated intracellular lipid metabolism. As illustrated in Figure 2a, *CH25H*, a key antiviral interferon-stimulated gene, encodes the multi-transmembrane endoplasmic reticulum (ER)-associated enzyme cholesterol 25-hydroxylase. CH25H catalyzes the conversion of cholesterol to 25-hydroxycholesterol (25-HC) [76]. Upon stimulation with Toll-like receptor (TLR) ligands or IFN-I, macrophages and dendritic cells significantly upregulate the expression of CH25H [77]. As a broad-spectrum antiviral effector, 25-HC exerts multiple antiviral effects, including restricting the fusion of viruses with host cell membranes, and inhibiting viral replication and assembly [76]. Specifically, 25-HC impairs viral adsorption, entry, and release by modulating cholesterol metabolism [76]. Since the adsorption and entry of certain viruses preferentially occur in lipid rafts of the cell membrane, 25-HC can embed into the cell membrane and reduce its cholesterol content, thereby disrupting the integrity and stability of cholesterol-enriched membrane domains and directly inhibiting viral adsorption as well as membrane fusion of enveloped viruses [76]. Mechanistically, 25-HC binds to insulin-induced gene 2 (INSIG2) protein, which in turn suppresses the activation of SREBP2 and thus inhibits de novo cholesterol synthesis [78]. Meanwhile, as an endogenous ligand for LXRs, 25-HC activation upregulates the expression of cholesterol transporters, promoting cholesterol esterification and storage in lipid droplets [79]. This synergistic response limits the availability of free cholesterol and other lipid components that are essential for efficient viral replication. For instance, 25-HC inhibits the membrane fusion of Zika virus (ZIKV) with host cell membranes, and alleviates viremia and fetal microcephaly in ZIKV-infected mouse and rhesus monkey models [80]. Certain RNA viruses rely on oxysterol-binding proteins (OSBPs) and OSBP-related proteins (ORPs) to transport cholesterol from the Golgi apparatus and ER membranes to replication sites within host cell membranes [81]. By binding to OSBPs and ORPs, 25-HC blocks the inter-organellar trafficking of cholesterol, thereby inhibiting the replication of these viruses [81]. A case in point is rhinovirus infection, where 25-HC prevents OSBP-mediated cholesterol delivery to viral replication membranes, consequently suppressing viral RNA replication [82]. The above process exemplifies a typical strategy whereby the IFN pathway actively suppresses host anabolic metabolism to restrict viral replicative resources. Specifically, IFN-I coordinately inhibit the expression of genes involved in de novo cholesterol and fatty acid synthesis (e.g., *Srebp2*, *Hmgcr*) in macrophages [83,84], thereby depriving the virus of essential endogenous lipid precursors [83]. Research by Blanc et al. demonstrated that stimulation with IFN-β or viral infection leads to a coordinated downregulation of genes across the entire sterol biosynthesis pathway and reduces intracellular free cholesterol levels [84]. This effect is strictly dependent on IFNAR1 signaling [84]. Importantly, pharmacological inhibition of this pathway using statins can mimic the protective effect of IFN and effectively suppress viral replication [84].

*RSAD2* (also known as cytomegalovirus-induced gene 5, *CIG5*) encodes the ER-associated protein viperin, which has been demonstrated to inhibit the fusion, replication, assembly, and budding of multiple viruses by downregulating the lipid metabolic pathway [85]. The expression of *RSAD2* is primarily regulated by IFN-I and IFN-II through the classical JAK/STAT signaling pathway [29,86]. The structure of viperin includes: a variable N-terminal domain featuring an amphipathic α-helix and a leucine zipper motif; a conserved central domain containing a radical S-adenosylmethionine (SAM) domain; and a highly conserved C-terminal domain [87]. The N-terminal domain anchors viperin to the endoplasmic reticulum and lipid droplets (LDs), a localization critical for its antiviral function [87]. The SAM domain is the core catalytic activity of viperin, involved in the regulation of lipid metabolism and viral inhibition [87]. Based on this domain, viperin catalyzes the synthesis of 3′-deoxy-3′,4′-didehydro-CTP (ddhCTP) from CTP, thereby directly limiting viral replication [87,88]. In recent years, it has been demonstrated that the C-terminal domain of viperin can activate tumor necrosis factor receptor-associated factor 6 (TRAF6) -dependent ubiquitination signaling and enhance the IFN-I response [89,90]. As shown in Figure 2a, viperin inhibits cholesterol biosynthesis through its SAM domain [91], thereby interfering with the replication of multiple enveloped viruses [92]. Additionally, viperin functions by binding to and inhibiting the activity of farnesyl pyrophosphate synthase (FPPs), a key enzyme responsible for synthesizing isoprenoid-derived lipids [26]. This interaction alters the structure of plasma membrane lipid rafts and reduces membrane fluidity, thereby inhibiting the release of Influenza A virus (IAV) from the host cell membrane [26]. Beyond membrane properties, lipid metabolism also influences viral assembly through LDs, which serve as crucial platforms for virion assembly and provide energy substrates for the virus [93,94]. Viperin has been reported to disrupt the formation of LDs in host cells, thereby inhibiting viral assembly and budding [95]. Collectively, the interferon signaling pathway restricts viral replicative resources by modulating host anabolic metabolism directly or indirectly.

## 5. ISG-Mediated Remodeling of Amino Acids and Nucleotide Metabolism

Amino acids and nucleotides serve not only as the fundamental building blocks for proteins and nucleic acids but also as critical players in energy supply, signal transduction, and immune responses [96]. Amino acids can act as alternative carbon sources entering the tricarboxylic acid (TCA) cycle to maintain metabolic homeostasis and serve as precursors for the synthesis of essential molecules such as purines and pyrimidines, thereby contributing to redox balance [97]. Furthermore, specific amino acids directly regulate immune responses: for example, NO produced from arginine metabolism activates macrophages and promotes pathogen clearance, while tryptophan catabolism suppress T cell function via the kynurenine pathway [98,99,100].

Nucleotide metabolism similarly occupies a central position in cellular physiology [101]. Nucleoside triphosphates, such as ATP and GTP, serve as universal energy carriers [102]. Cyclic nucleotides, including cyclic adenosine monophosphate (cAMP) and cyclic guanosine monophosphate (cGMP), act as key second messengers in signal transduction [103]. Cofactors such as NAD^+^, NADP^+^, and flavin adenine dinucleotide (FAD) drive essential redox reactions [104,105,106]. To support the rapid proliferation of immune cells during viral infection, nucleotides are supplied through either de novo synthesis or salvage pathways [107]. Meanwhile, fluctuations in intracellular deoxynucleoside triphosphate (dNTP) levels can activate the cGAS–STING pathway, inducing the production of IFN-I, which constitutes a key step in viral sensing and antiviral defense [108].

### 5.1. Reprogramming of Amino Acid Metabolism by Viruses and ISGs

Viral infection extensively reshapes host amino acid metabolism [60]. For instance, vaccinia virus (VACV) replication relies on asparagine [109]. Glutamine serves as both an energy source for immune cells and a modulator of ISG expression, thereby enhancing IFN-mediated antiviral responses [110,111]. IAV has been shown to significantly alter host glutamine and tryptophan metabolic pathways, which in turn drives functional changes in immune cells and shapes inflammatory responses [112,113]. In response, the host actively remodels amino acid metabolism to bolster antiviral defenses. One prominent example is the production of NO from arginine via nitric oxide synthase (NOS), which directly restricts viral replication while simultaneously enhancing the IFN-I response [114].

Notably, IFN signaling actively reprograms amino acid metabolic pathways by inducing the expression of specific enzymes. A classic ISG-encoded protein, indoleamine 2,3-dioxygenase 1 (IDO1), catalyzes the degradation of tryptophan via the kynurenine pathway [115,116]. IDO1 is markedly upregulated in response to both IFN-I and IFN-II signaling, leading to tryptophan depletion and limiting substrate availability for viral replication [116]. Furthermore, the resulting kynurenines can activate the aryl hydrocarbon receptor (AhR), promoting the production of immunosuppressive factors such as interleukin-10 (IL-10) and transforming growth factor-beta (TGF-β), which helps restrain excessive inflammation [117,118]. However, prolonged AhR activation may also suppress IFN-I signaling and potentially contribute to viral persistence [119].

### 5.2. Modulation of Nucleotide Metabolism in Virus–Host Interactions

Nucleotide metabolism likewise undergoes extensive remodeling during virus–host interactions [120]. Viruses hijack host purine and pyrimidine synthesis pathways to support their own replication [120]. For example, SARS-CoV-2 has been shown to exploit the host pyrimidine biosynthetic pathway [121]. In contrast, the host utilize nucleotide-derived signaling molecules to activate antiviral immunity. A key mechanism is the catalysis of cGAMP by cGAS, which activates the STING-TBK1-IRF3 axis to induce IFN-I production, a process crucial for combating viral infection [122].

ISGs also participate in immune responses by modulating nucleotide metabolism. For example, *CD38* encodes a protein that promotes immune cell activation and initiates antiviral defense in early infection by regulating NAD^+^ metabolism and calcium ion (Ca^2+^) signaling [123,124]. As a multifunctional enzyme, CD38 hydrolyzes NAD^+^ into ADP-ribose (ADPR) and nicotinamide and also functions as an ADP-ribosyl cyclase to synthesize cyclic ADP-ribose (cADPR) [123,125]. cADPR promotes Ca^2+^ release from the endoplasmic reticulum, thereby facilitating immune cell activation, proliferation, and cytokine production [125]. However, sustained high expression of CD38 leads to decreased intracellular NAD^+^ levels, impairing glycolysis and mitochondrial function [126]. This mitochondrial dysfunction exemplifies the complexity of metabolic networks, where a single perturbation in NAD^+^ homeostasis can cascade into broader bioenergetic and signaling failures. This metabolic compromise results in cellular energy insufficiency, contributing to an exhausted immune phenotype [127], while excessive Ca^2+^ release may exacerbate inflammatory responses and tissue damage [128].

In summary, amino acid and nucleotide metabolism are also dynamically reshaped during viral infection, serving as a crucial interface between host defense and viral manipulation. ISGs act as central mediators of these metabolic reprogramming, enhancing antiviral immunity through diverse mechanisms.

## 6. Viral Hijacking of ISG-Mediated Metabolic Dysregulation in Immune Evasion and Immunopathology

In the evolutionary arms race between virus and host, the functions of ISGs are not immutable. Whether they act in a “protective” or “pathogenic” manner depends not on any intrinsic property of the genes themselves, but entirely on their context-dependent functional output. As previously mentioned, many protective ISGs inhibit viral infection or alleviate tissue damage caused by excessive immune activation by regulating host cell metabolism. Nevertheless, viruses have also evolved various mechanisms to hijack ISGs, thereby remodeling cell metabolism, achieving immune evasion, and modulating inflammatory responses, ultimately promoting their own infection or exacerbating pathological damage (Figure 2b). Crucially, this functional “switch” highlights how viruses subvert the host’s immunometabolic defenses for their own benefit, which is central to understanding the diversity of infections and complex disease pathologies. It is particularly noteworthy that the pathogenic ISGs discussed here share a common theme: they ultimately drive disease pathogenesis by hijacking or disrupting host metabolic homeostasis, such as mitochondrial function and lipid balance.

For example, the HCMV encodes the viral mitochondrial inhibitor of apoptosis (vMIA), which hijacks the ISG product viperin to facilitate infection [11]. Normally localized to the endoplasmic reticulum, viperin is redirected to mitochondria via an N-terminal mitochondrial targeting signal carried by vMIA. Within mitochondria, viperin binds to hydroxyacyl-CoA dehydrogenase trifunctional multienzyme complex subunit beta (HADHB), the β-subunit of the mitochondrial trifunctional enzyme complex. This interaction not only suppresses the thiolase activity of the enzyme and impairs the fatty acid β-oxidation process, but also induces retrotranslocation of HADHB, ultimately leading to its proteasomal degradation. The process leads to decreased ATP and NADH levels and disrupts the actin cytoskeleton, thereby creating favorable conditions for viral replication. Notably, HADHB itself can activate viperin to catalyze the synthesis of the antiviral nucleotide ddhCTP, underscoring a complex regulatory relationship between them [11]. Recent work further demonstrates that the 3C protease of CVB3 inhibits viperin degradation by downregulating the ubiquitination factor UBE4A, leading to abnormal viperin accumulation [129]. Excess viperin then interacts with STAT1 and promotes its degradation, which in turn activates the SGK1-KCNQ1 signaling axis, ultimately contributing to cardiac electrical dysfunction and acute heart failure [129]. These examples illustrate how viruses precisely hijack specific ISGs to reprogram the complex metabolic and signaling networks of host cells, including by inducing mitochondrial dysfunction as a key pathological node, thereby driving viral replication and mediating tissue damage.

As noted earlier, ISG15 regulates glucose and lipid metabolism through ISGylation to restrict viral infection or mitigate tissue injury from immune hyperactivation [52]. To evade this mechanism, viruses have developed multiple counterstrategies, including inducing the transcription of ISG15 deconjugating enzymes, sequestering ISG15-modified viral proteins, directly suppressing ISG15 expression, and producing viral proteases with deISGylating activity [130]. For instance, the papain-like protease (PLpro) encoded by SARS-CoV-2 specifically cleaves ISG15 modifications from host proteins, thereby weakening its antiviral effect [131]. Interestingly, bat ISG15, due to amino acid differences at key sites, is resistant to cleavage by PLpro, which may be an important reason why bats can carry viruses without developing disease [132]. This provides a clear example of how viruses hijack the ISGylation system.

Other ISGs may likewise be exploited by viruses to alter host metabolic states. For example, oligoadenylate synthase-like (OASL) protein interacts with ribosomes to preferentially translate mRNAs involved in lipid metabolism, promoting fatty acid synthesis and accumulation [133]. During infection, this mechanism may be hijacked by viruses to enrich metabolites required for replication. Additionally, the phospholipid scramblase PLSCR1 inhibits viral entry, replication, and budding by maintaining membrane phospholipid asymmetry [134,135,136], yet multiple viruses have been shown to directly target PLSCR1 and disrupt its function.

Beyond mediating immune evasion, viruses also exacerbate immunopathological damage by hijacking ISGs to reprogram host metabolism. IAV infection, for example, can trigger a cytokine storm linked to excessive pro-inflammatory signaling and inadequate anti-inflammatory control [12]. It has been shown that IAV infection redirects cellular glucose metabolism toward the hexosamine biosynthesis pathway, enhancing O-GlcNAc signaling. O-GlcNAc transferase (OGT) mediates O-GlcNAcylation of the IRF5 at residue S430, promoting its K63-linked ubiquitination and ultimately driving excessive expression of inflammatory cytokines. This indicates that IRF5, as an interferon regulatory factor, is hijacked during IAV infection to participate in mediating cytokine storms and tissue damage [12].

In summary, by precisely targeting ISGs and their regulated metabolic and signaling pathways, viruses not only evade host defenses but also actively reprogram cellular physiology to support replication, and in some cases exacerbate immune-mediated pathology. These mechanisms reveal the complex interplay between viruses and the host at the metabolism–immune interface and also offer potential targets for intervention strategies against virus-related immunopathology.

## 7. Conclusions and Future Perspectives

This review elucidates that ISGs exert antiviral effects through the reprogramming of host cell metabolism, including the regulation of glucose, lipid, amino acid, and nucleotide metabolism. This expands our understanding of ISG mechanisms and host defense strategies. Furthermore, we propose the dual nature of ISGs in antiviral infection: while most ISGs function as core antiviral effectors protecting the host against infection, a subset may be hijacked by viruses to instead promote viral infection or exacerbate inflammatory responses, thereby contributing to viral immune evasion and pathogenesis. Understanding the roles of “protective ISGs” versus “pathogenic ISGs” in virus–host interactions from a metabolic perspective is likely an emerging frontier in virology.

Building on the protective role of ISG15 in a CVB3-induced myocarditis model [5], future research could explore manipulating metabolic shifts mediated by such “protective ISGs” (e.g., a switch from glycolysis to oxidative phosphorylation) to develop novel strategies for ameliorating virus-associated cardiac injury. Conversely, identifying key “pathogenic ISGs” that mediate chronic viral infection and excessive inflammatory tissue damage will improve our understanding of the underlying pathological mechanisms. Developing antiviral therapeutic strategies targeting viral resistance mechanisms against these key “pathogenic ISGs” holds promise for effectively inhibiting viral immune evasion and mitigating host damage.

Importantly, as central hubs integrating immune and metabolic signaling, the regulatory networks of ISGs revealed in the context of viral infection provide a critical paradigm for understanding their roles in chronic diseases. For instance, in the tumor microenvironment, proteins encoded by ISGs, such as ISG15, OASL, and viperin, drive glycolysis and/or lipid metabolism reprogramming [133,137,138,139], directly supporting tumor growth and progression. In autoimmune diseases like systemic lupus erythematosus (SLE), chronic IFN stimulation shapes a persistent epigenetic memory for ISG expression through metabolites like α-ketoglutarate (α-KG) [140]. Additionally, viperin has been reported as a pathogenic ISG that contributes to pregnancy abnormalities in SLE patients by promoting lipid accumulation at the maternal–fetal interface [141]. During aging, the upregulation of ISG15 in the ovary contributes to age-related infertility by degrading a key follicular maturation protein, driving ovarian inflammation, and accelerating cellular senescence [142]. Collectively, this evidence indicates that ISG-mediated metabolic remodeling represents a common thread linking acute infection, chronic inflammation, and the pathogenesis of various diseases.

To dissect the cell-type-specific functions and metabolic networks of ISGs within the complex architecture of infected or diseased tissues, future studies should leverage integrated spatial multi-omics approaches. Combining spatial transcriptomics with spatial metabolomics can map, at single-cell or near-single-cell resolution, the precise spatial correlations between cellular states, ISG expression, and local metabolite levels in situ. This will be pivotal for understanding how ISG-driven metabolic reprogramming differentially shapes immune and stromal cell fates within specific tissue microenvironments. Subsequently, further exploration of novel therapeutic strategies that target these specific metabolic pathways (e.g., intervening in the ISG15-associated energy metabolic shift in CVB3 myocarditis) to ameliorate immunopathology is warranted [5]. Future studies should also aim to dissect potential metabolic reprogramming differences mediated by ISG subsets preferentially induced by specific interferon types (e.g., type I vs. type II), which may reveal finer regulatory layers of immunometabolic control. Concurrently, investigating the mechanisms evolved by viruses to escape ISG-mediated metabolic defenses will provide new targets for developing broad-spectrum antiviral agents. Ultimately, a profound understanding of the dynamic interplay between virus and host at the metabolic level will not only advance virology itself but also holds transformative potential for the treatment of cancer, autoimmune diseases, and aging-related disorders across disciplinary boundaries.

## Data Availability

No new data were created in this study. All data supporting the findings of this research are derived from published literature, and the relevant citations are fully provided within the paper.
